# Lack of viral clearance by the combination of hydroxychloroquine and azithromycin or lopinavir and ritonavir in SARS-CoV-2-related acute respiratory distress syndrome

**DOI:** 10.1186/s13613-020-00678-4

**Published:** 2020-05-24

**Authors:** Sami Hraiech, Jérémy Bourenne, Khaldoun Kuteifan, Julie Helms, Julien Carvelli, Marc Gainnier, Ferhat Meziani, Laurent Papazian

**Affiliations:** 1 Assistance Publique - Hôpitaux de Marseille, Hôpital Nord, Médecine Intensive Réanimation, Hôpital Nord, 13015 Marseille, France; 2 Faculté de médecine, Centre d’Etudes et de Recherches sur les Services de Santé et qualité de vie EA 3279, Aix-Marseille Université, Marseille, 13005 France; 3 Assistance Publique - Hôpitaux de Marseille, Hôpital Timone, Médecine Intensive Réanimation, 13005 Marseille, France; 4 Réanimation Médicale, GHR Mulhouse Sud Alsace, 68100 Mulhouse, France; 5 Service de Médecine Intensive – Réanimation, Nouvel Hôpital Civil, Hôpitaux universitaires de Strasbourg, Strasbourg, France

The current international outbreak of respiratory illness due to SARS-CoV-2 and named Covid-19 can evolve to severe progressive pneumonia and acute respiratory distress syndrome (ARDS), multiorgan failure, and death. Up to now, there are no specific therapeutic agents for coronavirus infections. Lopinavir–ritonavir treatment recently failed to demonstrate any significant outcome benefit, but the study was underpowered to rule out clinically meaningful treatment effects, and the intervention was started a median of 13 days after symptoms onset [1]. In vitro inhibition of virus spread has been reported with chloroquine prior to or after SARS-CoV-2 infection [2]. Hydroxychloroquine has been found to be more potent than chloroquine to inhibit SARS-CoV-2 in vitro [3] and a recent report suggested that 70% of 20 non-ICU hydroxychloroquine-treated patients had negative PCR results in nasopharyngeal samples at day 6 (D6) post-inclusion [4] and in all the 6 patients treated with hydroxychloroquine and azithromycin combination (hydroxychloroquine–azithromycin) [4]. In order to evaluate these results in intensive care unit (ICU) patients, we retrospectively assessed in moderate-to-severe ARDS the efficacy of hydroxychloroquine–azithromycin combination regarding viral disappearance at both day 6 of the treatment and day 6 of evolution of ARDS as compared with patients treated with lopinavir–ritonavir and a control group without any anti-viral treatment.

Forty-five patients were included, 17 receiving the combination of hydroxychloroquine 600 mg and azithromycin 500 then 250 mg daily, 13 receiving lopinavir–ritonavir 800 mg daily and 15 who did not receive any anti-viral treatment (controls). Patients were admitted to 4 ICUs in 2 different regions of France from March 2nd to March 31st. In one ICU, they received hydroxychloroquine–azithromycin as a usual policy while this combination was maintained if started prior to admission in the second ICU (the other patients receiving lopinavir–ritonavir). Controls were treated in 2 other ICUs with antibiotics targeting bacterial community acquired pneumonia only. In all patients, nasopharyngeal PCR for SARS-CoV-2 were performed at the time of diagnosis and then regularly during ICU stay in order to assess viral clearance. Results of PCR were qualitative at the beginning of the pandemic, then quantitative and expressed by the PCR cycle threshold (CT).

Data were expressed as mean ± the standard deviation or median with interquartile range for the quantitative variables, and as numbers and percentages for the categorical variables. Groups were compared using the Chi-square or Fisher’s exact test for categorical characteristics, and using the Student’s *t* test or Mann–Whitney U test for continuous ones. A two-sided *p* value of less than 0.05 was considered statistically significant.

Results are displayed in Table 1. Patients presented ARDS criteria 2 ± 5 days after diagnosis confirmation and 1 ± 2 days after treatment onset. Negative nasopharyngeal PCR for SARS-CoV-2 at day 6 following the initiation of treatment were observed in 5 (38%) patients from the lopinavir–ritonavir group as compared with 3 (18%) patients from the hydroxychloroquine–azithromycin group and 2 (20%) from the control group (*p* = 0.39). At day 6 following ARDS onset, PCR was negative in only 9 patients, 5 from the lopinavir–ritonavir group, 2 from the hydroxychloroquine–azithromycin group and 2 from the control group. When considering only the patients that had received an anti-viral treatment within the 5 days following the onset of COVID-19 symptoms, we found that none of them (0/7) had a negative PCR 6 days after the beginning of treatment in the hydroxychloroquine–azithromycin group as compared with 3/7 (43%) in the lopinavir–ritonavir group (*p* = 0.05). At day 6 following ARDS, mortality was 4.4%, all survivors being under mechanical ventilation (MV) with no difference regarding ventilatory parameters, use of adjuvants and SOFA score. The latest follow-up done 38 ± 7 days following treatment onset revealed that 37 patients were still alive (82%), 12 (92%) in the lopinavir–ritonavir group, 15 (88%) in the hydroxychloroquine–azithromycin group and 10 (67%) in the control group. Ten patients were still in ICU, 6 (35%) from the hydroxychloroquine–azithromycin group, and 4 (31%) from the lopinavir–ritonavir group.

**Table 1:** Characteristics and main outcomes

	Hydroxychloroquine/azithromycin (*n* = 17)	Lopinavir/ritonavir (*n* = 13)	Controls (*n* = 15)	*p* value
Age (years, mean ± SD)	60 ± 17	62 ± 13	60 ± 16	0.94
Male gender	15 (88%)	9 (69%)	11 (73%)	0.41
Hypertension	8 (47%)	8 (62%)	5 (33%)	0.33
Diabetes mellitus	6 (35%)	5 (38%)	2 (13%)	0.26
Chronic cardiac disease	2 (12%)	5 (38%)	0	0.02
Chronic respiratory disease	2 (12%)	2 (15%)	1 (7%)	0.76
Obesity	6 (35%)	6 (46%)	9 (60%)	0.38
Immunosuppressive therapy	1 (6%)	1 (8%)	0 (0%)	0.58
Time from COVID-19 symptoms onset to treatment (days, median, IQR)	7 (4–11)	5 (2–6)	8 (4.5–10.5)^a^	0.06
Time from COVID-19 treatment to ARDS (days, median, IQR)	1 (0–4)	0 (0–0)	0 (0–0)^a^	0.02
SAPS 2 score (mean ± SD) at ICU admission	30 ± 7	33 ± 11	49 ± 14	< 0.01
SOFA score at ICU admission (median, 1st–3rd Quartile)	4 (3–6)	6 (3–7)	6 (4–9.5)	0.27
PaO2/FiO2 ratio at ICU admission (mean ± SD)	141 ± 48	159 ± 37	129 ± 50	0.21
Vt at ICU admission (ml, mean ± SD)	388 ± 64	392 ± 76	415 ± 42	0.59
PEEP at ICU admission, (cm H_2_O, median, 1st–3rd quartile)	12 ± 2	13 ± 2	13 ± 3	0.8
SARS-CoV-2 PCR CT values at diagnosis (mean ± SD)^b^	28 ± 5	25 ± 9	–	0.88
SARS-CoV-2 PCR CT values at day 6 from treatment (mean ± SD)^b^	29 ± 5	33 ± 1	–	0.01
Negative SARS-CoV-2 PCR at day 6 from treatment	3 (18%)	5 (38%)	2^c^ (20%)	0.39
Negative SARS-CoV-2-PCR at day 6 from ARDS	2 (12%)	5 (38%)	2 (13%)	0.14
PaO2/FiO2 ratio at day 6 from ARDS onset (mean ± SD)	160 ± 59	183 ± 64	150 ± 46	0.69
SOFA score at day 6 from treatment (median, IQR)	4.5 (3–6)	6 (4–6)	6 (5–7)	0.9
SOFA score at day 6 from ARDS onset (median, IQR)	4.5 (3–7)	6 (4–6)	6 (5–7)	0.9
Alive at day 6 from ARDS	15 (88%)	13 (100%)	15 (100%)	
Invasive mechanical ventilation^d^	16 (94%)	12 (92%)	15 (100%)	
Neuromuscular blockers^d^	16 (94%)	11 (85%)	14 (93%)	
Prone positioning^d^	14 (82%)	8 (62%)	12 (80%)	
Inhaled nitric oxide^d^	4 (24%)	3 (23%)	4 (27%)	
ECMO^d^	2 (12%)	1 (8%)	1 (7%)	

*ARDS* acute respiratory distress syndrome, *CT* cycle threshold, *ECMO* extracorporeal membrane oxygenation, *ICU* intensive care unit, *IQR* interquartile range, *PEEP* positive end-expiratory pressure, *SAPS 2* Simplified Acute Physiology Score, *SOFA* Sepsis-related Organ Failure Assessment score, *Vt* tidal volume

^a^Antibiotics (no anti-viral treatment and no azithromycin in this group)

^b^When available, in positive patients

^c^Only 10 patients assessed

^d^During the first 6 days of ARDS

- Data not available

In this case–control study, the rates of viral clearance at day 6 after treatment were not significantly different between patients treated with hydroxychloroquine and azithromycin, patients treated with lopinavir–ritonavir and those not treated with any specific anti-viral treatment. No difference in SARS-CoV-2 PCR negativity was found between groups 6 days after meeting moderate-to-severe ARDS criteria. Groups were comparable, except for a higher severity at admission in control patients, who were more frequently transferred to the ICU only when requiring MV, because of the massive influx of patients in this region of France. Although a positive PCR is not synonymous with active viral development, these results highlight the fact that neither of the treatments was able to achieve a rapid viral clearance in ARDS patients, as it has been suggested in one report on non-severe patients [4]. Waiting for the results of ongoing randomized controlled trials, clinicians should prescribe these treatments taking into account the paucity of the current rationale and the risk–benefit ratio in severe forms. Moreover, considering the lack of viral clearance in the most severe patients, the use of immunosuppressive drugs should be carefully balanced in this population [5].

## Data Availability

The datasets used and/or analysed during the current study are available from the corresponding author on reasonable request.
